# Hybrid extended Kalman filter with Newton Raphson method for lifetime prediction of lithium-ion batteries

**DOI:** 10.1038/s41598-025-91156-z

**Published:** 2025-04-26

**Authors:** Hend M. Fahmy, Hany M. Hasanien, Mohammed Alharbi, Haoran Ji

**Affiliations:** 1 Electrical Power and Machines Department, Faculty of Engineering, Ain Shams University, Cairo, 11517 Egypt; 2 Electrical Engineering Department, College of Engineering, King Saud University, Riyadh, 11421 Saudi Arabia; 3 The Key Laboratory of Smart Grid of Ministry of Education, Tianjin University, Tianjin, 300072 China; 4 Faculty of Engineering and Technology, Future University in Egypt, Cairo 11835, Egypt

**Keywords:** Extended Kalman filter, Lifetime prediction, Lithium-ion battery, Newton Raphson method, Energy science and technology, Electrical and electronic engineering

## Abstract

To advance the lithium-ion battery (LIB) technology more quickly, its lifetime should be predicted accurately. The precise prediction of LIB lifetime can help in producing new batteries, better use and operation of batteries. It is worthy for noting here that the LIB is a heavy nonlinear system suffering from battery fading, degradation, uncertainty and variability of operating conditions. Therefore, this article presents a hybrid extended Kalman filter with Newton Raphson method for lifetime prediction of lithium-ion batteries. The data analyses are based on commercial lithium iron phosphate/graphite cells cycled at fast charge. The cycle life expectancy is in the range of 150 to 2,300 cycles. The discharge voltage characteristics are used to present capacity degradation. The battery datasets are used with a hybrid Extended Kalman Filter (EKF) and Newton Raphson method to match the predicted cycle life and the actual cycle life of the battery. The effectiveness of the proposed method is verified by making a fair comparison with the linear regression-based machine-learning method. In the testing of 100 lifecycles, the test error and root mean square error record 3.26% and 10.93 compared with the linear regression that achieves 9.1% and 211, respectively. With the proposed hybrid approach, the lifetime prediction of LIBs can be further enhanced.

## Introduction

### Research problem

The desire for green technology, the high cost of fossil fuels, and various political disputes have led to a steady decline in the use of traditional energy sources^1^. Renewable energy resources, with all of their different types, are widely used in power systems. The majority of these resources have complex and sporadic power outputs. To handle these issues, various storage devices are integrated with these systems, improving systems reliability, stability, and overall efficiency^2–7^. It is thought that these storage devices are a potential technology for creating smart networks, incorporating electric vehicles and transportation systems^8^. Battery energy storage devices are considered widely used storage due to their affordable cost. Applications of battery-related energy storage devices are greatly impacted by battery performance, which is directly and critically determined by battery manufacture. Effective battery property forecasts and trustworthy analysis of strongly coupled battery production factors or variables become crucial but difficult concerns for broader battery applications because battery manufacture is a complex process that incorporates chemical, mechanical, and electrical activities^9^. These battery systems have different types based on manufacturing and technology. The Lithium-ion battery (LIB) has several merits, such as lower self-discharge rate, quick charging, higher efficiency, long lifetime, and higher energy density^10–15^. Therefore, it can be extensively used in various low and high-power applications such as microgrids, energy and transportation systems as well as electric vehicles^10,11,16^. To advance the LIB technology more quickly, its lifetime should be predicted accurately. The remaining useful life (RUL) is crucial to health management, problem diagnostics, and prediction during the equipment’s operational service duration^17^. The assumption for equipment to implement condition-based maintenance, fault maintenance, preventive maintenance, and other maintenance techniques is the RUL forecast result. One of the major tasks of lithium-ion battery management systems (BMS) is estimating the RUL. When a battery hits its end-of-life (EOL), its capacity rapidly declines, becoming more likely to fail, impacting equipment functioning and possibly resulting in safety incidents. Additionally, the user can change the battery too soon for safety reasons, which would squander battery resources. Thus, a significant and challenging issue is resource failure, which an accurate RUL forecast may prevent in addition to several safety incidents^18^. The precise prediction of LIB lifetime can help in producing new batteries, better use and operation of batteries^19,20^. However, this task is hard, where the LIB is a heavy nonlinear system suffering from battery fading, degradation, uncertainty, and variability of operating conditions^21–24^.

### Literature review

The continuous operation of LIBs through many charging and discharging cycles results in the degradation and fading of such batteries. Many abnormal conditions may be occurred like sudden faults^25,26^. Therefore, it is essential to predict the battery lifetime^27,28^. Several approaches have been proposed to model the battery lifetime. In^29^, semi-empirical modeling approaches are used for the power loss prediction. Moreover, many methods have been used to study the diverse mechanism^30^. The lifetime estimation models include direct measurements, prediction or data-driven, and equivalent circuits models^31^. Specific measurement methods such as Coulombic efficiency method and an impedance spectroscopy method are implemented in estimating the battery lifetime. These methods, which are carried out directly in the laboratory, depend on measurements of LIB characteristics like capacity and internal battery resistance^31^.

Furthermore, data-driven techniques are effectively applied to estimate the battery lifetime, excluding the intricate LIB model^32^. In these methods, the battery fade is determined by the battery characteristics. Machine learning strategies can be applied to match the predicted and actual health feature of batteries^33^. In^34^, the improved radial basis artificial neural network (ANN) is applied to estimate the LIB lifetime. In addition, the battery current can be predicted by a backpropagation ANN method^35^. The support vector regression is used for LIB lifetime estimation. The absolute error and the root mean square error (RMSE) are calculated for various models^36^. In^37^, various charging processes are done for LIB lifetime estimation. Moreover, an incremental capacity with detailed ANN technology are applied to obtain the target at different operating conditions^38^. In^39^, the long short-term memory (LSTM)-based deep learning method is proposed for the lifetime battery prediction. The bidirectional LSTM approach is further implemented in^40^. Although the methods mentioned above are effective, they have certain disadvantages such as lengthy procedures, complex models, and memory requirements. The LSTM is used with ANN structure to achieve robust estimation^41^. A singular filtering-Gaussian LSTM method is used for the battery lifetime estimation^42^. In^43^, a broad learning and vector machine method is presented to estimate LIB lifetime. The gated recurrent unit is also implemented in^44^. Statistical Data-Driven AI is widely used in SOH estimation, such as support vector machine (SVM)^45^, Gaussian process regression (GPR)^46^, and Bayesian network (BN)^47^; these methods are very effective, but due to its complex computation, it puts these methods in disadvantage. Sequence-to-sequence model with variational mode decomposition was used to predict the RUL for the lithium-ion battery^48^, this technique combines the variational modal decomposition with bi-directional long short-term memory and Bayesian hyperparametric optimization, this method gave a highly accurate RUL estimation of electric vehicles, but the computational complexity and computational time was very high.

The equivalent circuit-based model is applied to estimate state of charge and lifetime of lithium-ion batteries and various methods have been used for that target. Several optimization methods are applied for these battery estimation such as the particle swarm optimization^49^, the genetic algorithm^50^and others^51^. In^52^, the support vector machine approach is used prediction of battery state of charge. Moreover, the dual adaptive filters are applied for this battery estimation^53,54^. The sparse Bayesian learning approach and unscented Kalman filters are further used for lifetime prediction of LIBs^55–58^. Though these techniques are effective, there is still room for improvement in terms of estimating accuracy of battery lifetime.

### Principal contribution

To advance the LIB technology more quickly, its lifetime should be predicted accurately. The precise prediction of LIB lifetime can help in producing new batteries, better use and operation of batteries. It is worthy for noting here that the LIB is a heavy nonlinear system suffering from battery fading, degradation, uncertainty and variability of operating conditions. Therefore, this article presents a hybrid extended Kalman filter with Newton Raphson method for lifetime prediction of lithium-ion batteries. The data analyses are based on commercial lithium iron phosphate/graphite cells cycled at fast charge. The cycle lives are in the range of 150 to 2,300 cycles^30^. The discharge voltage characteristics are used to present capacity degradation. The battery datasets are used with the hybrid extended Kalman filter and Newton Raphson method to match the predicted cycle life and the actual cycle life of the battery. The effectiveness of the proposed method is verified by making a fair comparison with the linear regression-based machine-learning method. In the testing of 100 lifecycles, the test error and RMSE record 3.26% and 10.93 compared with the linear regression that achieves 9.1% and 211, respectively. With the proposed method, the lifetime prediction of LIBs can be further enhanced.

### Paper structure

In this article, Sect. 2 presents dataset generation and problem formulation. In Sect. 3, the proposed methodology is explained including extended Kalman filter, the Newton Raphson method, and linear regression-based machine learning method. Section 4 exhibits the numerical results and their verification with experimental results. In Sect. 5, conclusions are presented, and future works are stated.

## Data production and problem formulation

### Data production

**Fig. 1:**
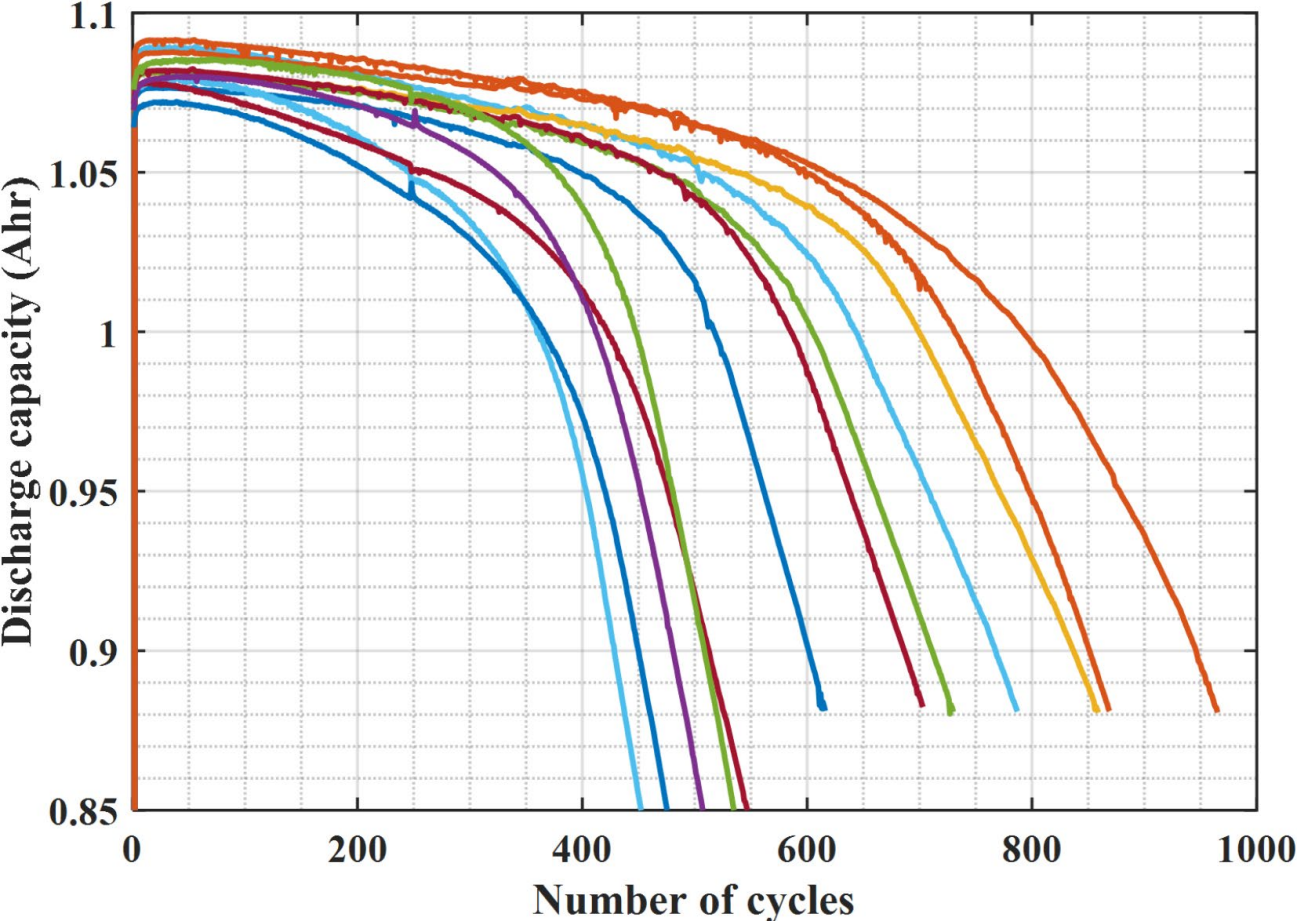
Discharge capacity with the number of cycles of Lithium-ion batteries.

The dataset includes commercial lithium-ion batteries that are fast-charged to failure^30^. These 48-channel Arbin LBT lithium-ion phosphate (LFP)/graphite cells from A123 Systems (APR18650M1A) are cycled in horizontal cylindrical fixtures under different fast-charging settings but the same discharging conditions^30^. The temperature is maintained at 30 °C using forced convection and batteries have a nominal voltage and capacity of 3.3 V and 1.1 Ah, respectively^30^. A wide variety of cycle durations, from about 150 to 2,300 cycles, are included in the dataset reported in detail in^30^. The datasets are based on 124 LIBs and are divided into three categories, including 41 cells for training, 43 cells for validation, and 40 cells for testing. Figure 1illustrates samples of the discharge capacity as a function of cycle number for the first 1,000 cycles^30^. As is common with lithium-ion batteries, capacity loss increases as the battery nears its end of life after being relatively small for the first 100 cycles.

### Problem formulation

The bounds of prediction accuracy and the cost-benefit analysis of acquiring more data streams are evaluated using the model. Two measures are used to evaluate the prediction performance: the average percentage error and the root-mean-square error (RMSE) with cycles. The RMSE can be represented as follows:

1 $$\:RMSE=\sqrt{\frac{1}{N}\sum\:_{i=1}^{N}{\left({x}_{i}-{\widehat{x}}_{i}\right)}^{2}}$$

where $\:{x}_{i}$ is the actual cycle life and $\:{\widehat{x}}_{i}$ is the predicted cycle life and $\:N$ is the total number of cells. Moreover, the average percentage error is defined by the following equation:

2 $$\:\%AvgError=\:\frac{1}{N}\sum\:_{i=1}^{N}\frac{|{x}_{i}-{\widehat{x}}_{i}|}{{x}_{i}}\times\:100$$

## Proposed methodologies

### Extended Kalman filter

The EKF is the KF’s extension for nonlinear systems. To approximate the nonlinear system with a linear time-varying system, the EKF approach performs a linearization operation at each time step. Next, the genuine nonlinear system is obtained by utilizing the linear time-varying system in a KF to generate an EKF. Like the KF, the EKF forecasts the real state with the lowest mean squared error using the measured input and output. It assumes that sensor noise and process noise are separate, zero-mean Gaussian noises. Although the nonlinear system is approximated using a linear time-varying system, the efficacy of EKF has been demonstrated in several studies^54^. The EKF methodology is presented as follows:

The state space equations of the lithium-ion batteries are presented using Eqs. (3) and (4).

3 $$\:\dot{x}=f\left(x,u\right)+w$$

4 $$\:y=g\left(x,u\right)+v$$

where $\:u\left(t\right)$ is the battery input current, $\:y\left(t\right)$ is the battery output voltage, and $\:x$ is denoted as $\:x={\left[{x}_{1},{x}_{2}\right]}^{T}$ where $\:{x}_{1}$ is the cycle life of the battery and $\:{x}_{2}$ is the battery discharge voltage. The terms $\:w$ and $\:v$ indicate errors brought on by parameter changes in addition to random disturbances. Equations (5) and (6) provide the functions $\:f(x,u)$ and $\:g(x,u)$.

5 $$\:f\left(x,u\right)=\left[\frac{\frac{u}{Q}}{-\frac{1}{RC}{x}_{2}+\frac{1}{C}u}\right]$$

6 $$\:g\left(x,u\right)={x}_{1}+{x}_{2}+{R}_{o}\times\:u+{\left[\frac{-1}{0}\right]}^{T}$$

where $\:f\left(x,u\right)$ is the process function and $\:g\left(x,u\right)$ is the measurement function, $\:Q$ is the battery capacity, and $\:R$ and $\:C$ are the battery resistance and capacitance. When the functions $\:f(x,u)$ and $\:g(x,u)$ are linearized by a first order Taylor-series expansion about the present operating point at each sample step, the model is said to be linearized as follows:

7 $$\:\delta\:\dot{x}={A}_{x}{\delta\:}_{x}+{B}_{k}{\delta\:}_{u}$$

8 $$\:\delta\:y={C}_{k}{\delta\:}_{x}+{D}_{k}{\delta\:}_{u}$$

9 $$\:{A}_{k}={\left.\frac{\partial\:f\left(x,u\right)}{\partial\:x}\right|}_{x\left(t\right),u\left(t\right)}=\left[\begin{array}{cc}0&\:0\\\:0&\:\frac{-1}{RC}\end{array}\right]$$

10 $$\:{B}_{k}={\left.\frac{\partial\:f\left(x,u\right)}{\partial\:u}\right|}_{x\left(t\right),u\left(t\right)}={\left[\begin{array}{cc}1&\:1\\\:Q&\:C\end{array}\right]}^{T}$$

11 $$\:{C}_{k}={\left.\frac{\partial\:g\left(x,u\right)}{\partial\:x}\right|}_{x\left(t\right),u\left(t\right)}=\left[\begin{array}{cc}k&\:1\end{array}\right]$$

12 $$\:{D}_{k}={\left.\frac{\partial\:g\left(x,u\right)}{\partial\:u}\right|}_{x\left(t\right),u\left(t\right)}={R}_{o},\,\:{R}_{o}=Internal\,resistance$$

The discretization of Eqs. (7–12) is presented as follows:

13 $$\:{x}_{k+1}={A}_{d}{x}_{k}+{B}_{d}{u}_{k}$$

14 $$\:{A}_{d}\approx\:E+{\tau\:}_{s}\times\:{A}_{k}$$

15 $$\:{B}_{d}\approx\:{\tau\:}_{s}\times\:{B}_{k}$$

16 $$\:{B}_{d}\approx\:{\tau\:}_{s}\times\:{B}_{k}$$

17 $$\:{C}_{d}\approx\:{C}_{k}$$

18 $$\:{D}_{d}={D}_{k}$$

where E is a unit matrix and $\:{\tau\:}_{s}$ is the sampling time. The steps of EKF methodology comprises three phases, which can be described as follows:

**Phase (1): Initialization**.

Given are the values of the starting state $\:{x}_{o}$, the covariance matrix P, the noise variance $\:{R}_{w}$ and $\:{Q}_{v}$ for k = 0.

- $\:\widehat{x}\left(0\mid0\right):$ Measurements at time step 0 are used to estimate the state at that point.
- $\:\widehat{P}\left(0\mid0\right)$: Measurements at time step 0 are used to create the state estimation error covariance matrix.

The method repeats two stages at each sample interval after initialization. First, the output, error covariance, and current state value are anticipated. Second, it adjusts the state estimate and error covariance by measuring the physical system’s output. The execution of these two phases is guided by the following equations, as demonstrated:

**Phase (2): Prediction**.

$\:For\:k=\text{1,2},\dots\:$, the state estimation and error covariance prediction computation are:

19 $$\:{\stackrel{-}{x}}_{k/k-1}=f({\stackrel{-}{x}}_{k\:/k-1},{u}_{k-1})$$

20 $$\:{P}_{k\:/k-1}={A}_{k}\times\:{P}_{k-1\:/k-1}\times\:{A}_{k}^{T}+{R}_{w}$$

**Phase (3): Correction**.

• Compute Kalman Filter gain:

21 $$\:{K}_{k}={P}_{k\:/k-1}\times\:{C}_{k}^{T}{\left[{C}_{k}\times\:{P}_{k\:\:/k-1}\times\:{C}_{k}^{T}+{Q}_{v}\right]}^{-1}$$

Update the state estimate and error covariance with measurements

22 $$\:{\stackrel{-}{x}}_{k/k}={\stackrel{-}{x}}_{k/k-1}+{K}_{k}\left[{y}_{k}-g\left({\stackrel{-}{x}}_{k\:\:/k-1},{I}_{k}\right)\right]$$

23 $$\:{P}_{k\:/k-1}={A}_{k}\times\:{P}_{k-1\:/k-1}\times\:{A}_{k}^{T}+{R}_{w}$$

24 $$\:{{E}_{k}=y}_{k}-g\left({\stackrel{-}{x}}_{k\:\:/k-1},{I}_{k}\right)$$

**Fig. 2:**
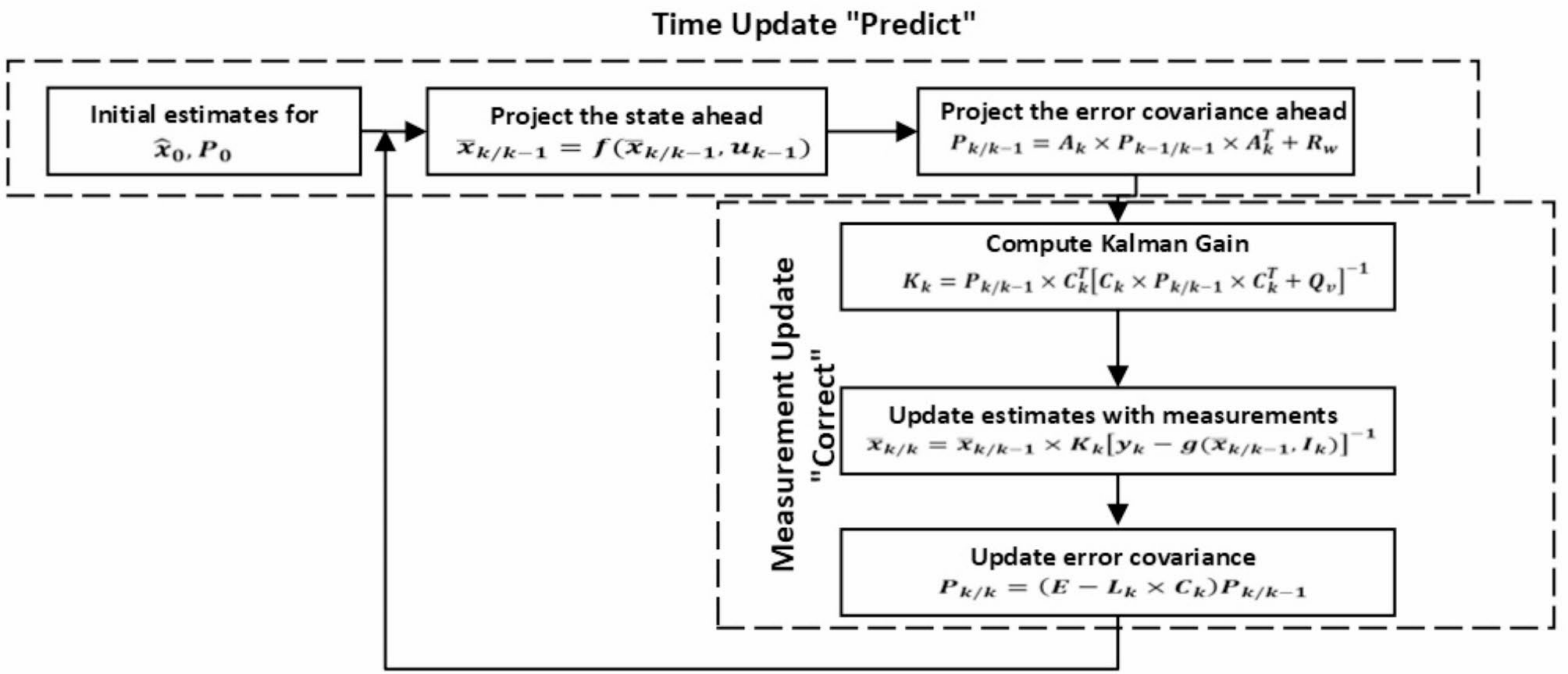
EKF Flowchart.

Start at the prediction phase and set k = k + 1 in order to keep predicting and correcting until optimum values are reached. Variations in the parameters can lead to mistakes in the EKF, which can be interpreted as noise in the system when predicting the cycle life of lithium-ion batteries. To summarize these procedures, the EKF flowchart is shown in Fig. 2.

### The Newton Raphson method

One particular use of the fixed-point iteration approach is the Newton-Raphson method^48^. Given an equation f (x) = 0, an initial approximation $\:{x}_{0}$ and the formula may be used to generate a series $\:{x}_{0},\:{x}_{1}$, .

25 $$\:{x}_{i+1}={x}_{i}-\frac{f\left({x}_{i}\right)}{\stackrel{\prime }{f}\left({x}_{i}\right)}$$

The approach will often converge when the initial point is in the vicinity of the zero and $\:f\:{\prime\:}\left(x\right)\:\ne\:\:0$ (and the convergence is at least quadratic if the zero has multiplicity 1).

It is demonstrated that the Newton-Raphson technique yields quadratic convergence if f (x) is sufficiently regular:

26 $$\:{g}_{n+1}\le\:d\:{g}_{n}^{2}\:with\:d\approx\:\frac{1}{2}\left|\frac{\stackrel{\prime}{\stackrel{\prime}{f}\left({x}^{*}\right)}}{\stackrel{\prime }{f}\left({x}^{*}\right)}\right|$$

where $$\:{g}_{n}\:=\:|{x}_{n}\:-\:{x}^{*}|$$

In other terms a good convergence is ensured when the constant $\:k$ is small and $\:{g}_{n}\:\ll\:\:1$.

### Linear regression

Severson et al.^24^ state that the cycle life of a lithium-ion battery is predicted using the machine learning linear regression approach; model fitting, or figuring out the coefficient values, and model selection, or figuring out the model structure, are both carried out. To do these jobs all at once, a regularization strategy is employed. The linear model of the form is shown as follows:

27 $$\:\widehat{{a}_{i}}=\widehat{s}\times\:{b}_{i}$$

where $\:s\:$is a p-dimensional model coefficient vector, $\:{b}_{i}$ is a p-dimensional feature vector for battery $\:i$, and $\:\widehat{{a}_{i}}$ is the anticipated number of cycles for battery i.

The least-squares optimization formulation includes a penalty factor to minimize overfitting when employing regularization techniques. The lasso and elastic net regularization methods simultaneously perform model fitting and selection by locating sparse coefficient vectors. It can be mathematically formulated by the following expression:

28 $$\:\widehat{s}=m{in}_{s}\times\:{\left|\left|a-Bs\right|\right|}_{2}^{2}+\gamma\:\times\:F\left(s\right)\:$$

where $\:B$ is the $\:N\:\times\:\:f$ feature matrix, $\:\gamma\:$ is a non-negative scalar, $\:a$ is the n-dimensional vector of observed battery lives, and the $\:min$ function represents determining the value of $\:s$ that minimizes the argument. The first term $\:{\left|\left|a-Bs\right|\right|}_{2}^{2}$, exists in regular least squares. The regularization method being used determines how the second term, $\:F\left(s\right)$, is expressed. For the lasso, it is expressed in Eq. (28) and as for elastic net, it is expressed in Eq. (29)

29 $$\:F\left(s\right)={\left|\left|s\right|\right|}_{1}$$

30 $$\:F\left(s\right)=\frac{1-\beta\:}{2}\times\:{\left|\left|s\right|\right|}_{2}^{2}+\beta\:\times\:{\left|\left|s\right|\right|}_{1}$$

where $\:\beta\:$ is a scalar number between 0 and 1.

## Simulation results

**Fig. 3:**
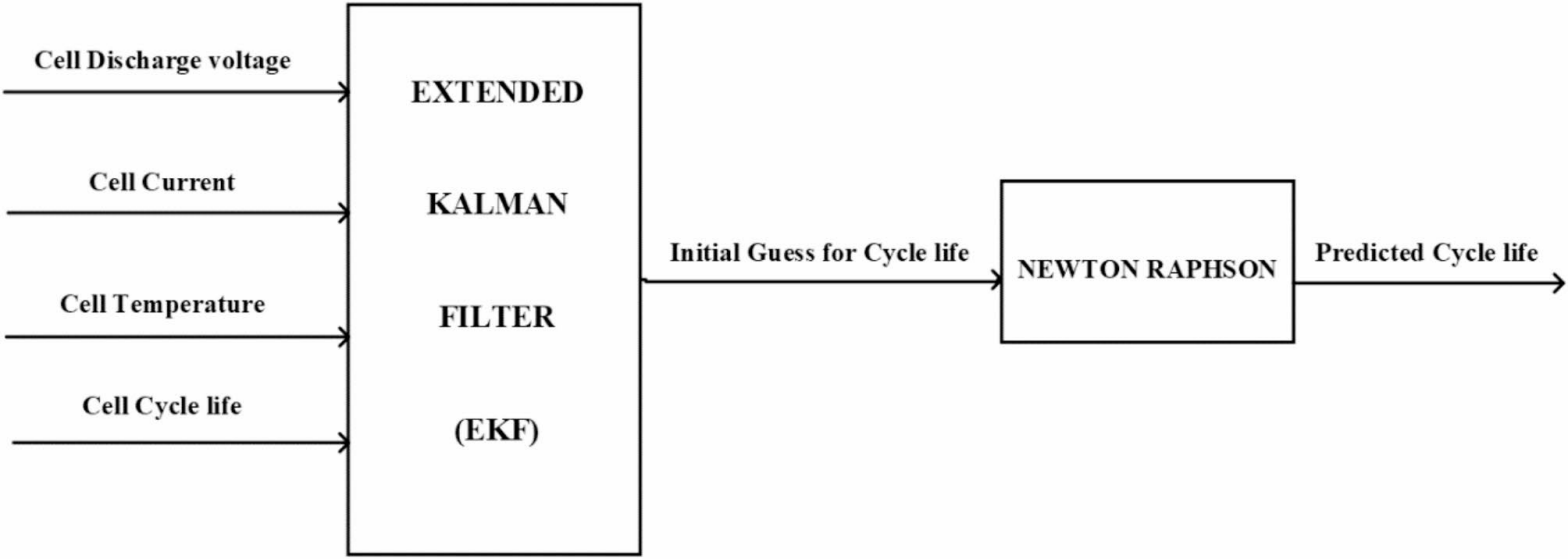
Schematic block diagram of the proposed method.

For lithium-ion batteries to operate as efficiently and last as long as possible, especially when used in electric cars and renewable energy storage, it is imperative to predict their cycle life. Through precise estimation of the number of charge-discharge cycles that a battery can withstand before seeing a substantial decline in capacity, scientists and producers may improve battery design, management tactics, and overall system performance. The prediction of batteries lifetime can help in obtaining batteries that are more long-lasting and robust, which in turn promotes cost-effectiveness, environmental sustainability, and better resource allocation. Furthermore, accurate cycle life forecasts enable customers to make well-informed choices about the lifetime and financial sustainability of battery-operated products, which eventually promotes the broad adoption of sustainable energy technology^30^. The suggested hybrid extended Kalman filter with Newton Raphson method is used to predict the remaining cycle life of lithium-ion battery with a minimal error between the measured and predicted data. 124 commercial LFP/graphite cells were cycled in a temperature-controlled environmental chamber (30 °C) under varied fast charging conditions but identical discharging conditions with a nominal capacity 1.1 Ahr and output voltage 3.3 V^30^. In this regard, the discharge voltage, current, temperature and cycle life are the main data feeding the EKF, then the output of the EFK is fed as an input to the Newton Raphson approach. The output signal of the hybrid method represents the data of predicted cycle lifetime of the battery. The schematic diagram of the proposed methodology is demonstrated in Fig. 3. The main features or characteristics of the lithium-ion battery are listed in Table 1. Table 2 illustrates the optimal tuning parameters of the EKF.

**Table 1:** Features of LFP/graphite lithium-ion battery.

Parameter	LFP/graphite cells
Number of cells	124
Nominal voltage (V)	3.3
Nominal capacity (Ahr)	1.1
Temperature (°C)	30 $\:\pm\:$10°C
Cell discharge voltage (V)	4 C = 2 V (where C = 1.1 A)
Charging time (min.)	10
Number of cycles	2,300

**Table 2:** Tuning parameters of EKF.

Filter	EKF
Initial state error covariance (P_0_)	[2, 0]
Measurement noise covariance (R)	0.01
Process noise covariance (Q)	[0.8, 0.2]

The proposed hybrid extended Kalman filter with Newton Raphson method is compared with the machine learning linear regression with elastic net regularization that are reported in detail in^24^. Two performance measurements criteria are used in comparison with the two techniques, the root-mean-square error (RMSE) with cycles and the average percentage error. Tables 3 and 4 demonstrate the RMSE and average percentage error value comparison between the proposed hybrid and the machine learning linear regression methodologies. As shown in the values of RMSE and average percentage error, the suggested hybrid methodology shows promising results as the RMSE value is reduced from 211.6148 using the machine learning linear regression to 10.9333 using the hybrid extended Kalman filter with Newton Raphson method and the average percentage error is reduced from 9.9817% using the linear regression to 3.2614% using the proposed method.

**Table 3:** RMSE values of proposed hybrid method and linear regression method.

	hybrid extended Kalman filter with Newton Raphson method	Linear regression
RMSE	10.9333	211.6148 [26]

**Table 4:** Average percentage error values of proposed method and linear regression method.

	hybrid extended Kalman filter with Newton Raphson method	Linear regression
Average Percentage Error	3.2614%	9.9817% [26]

**Fig. 4:**
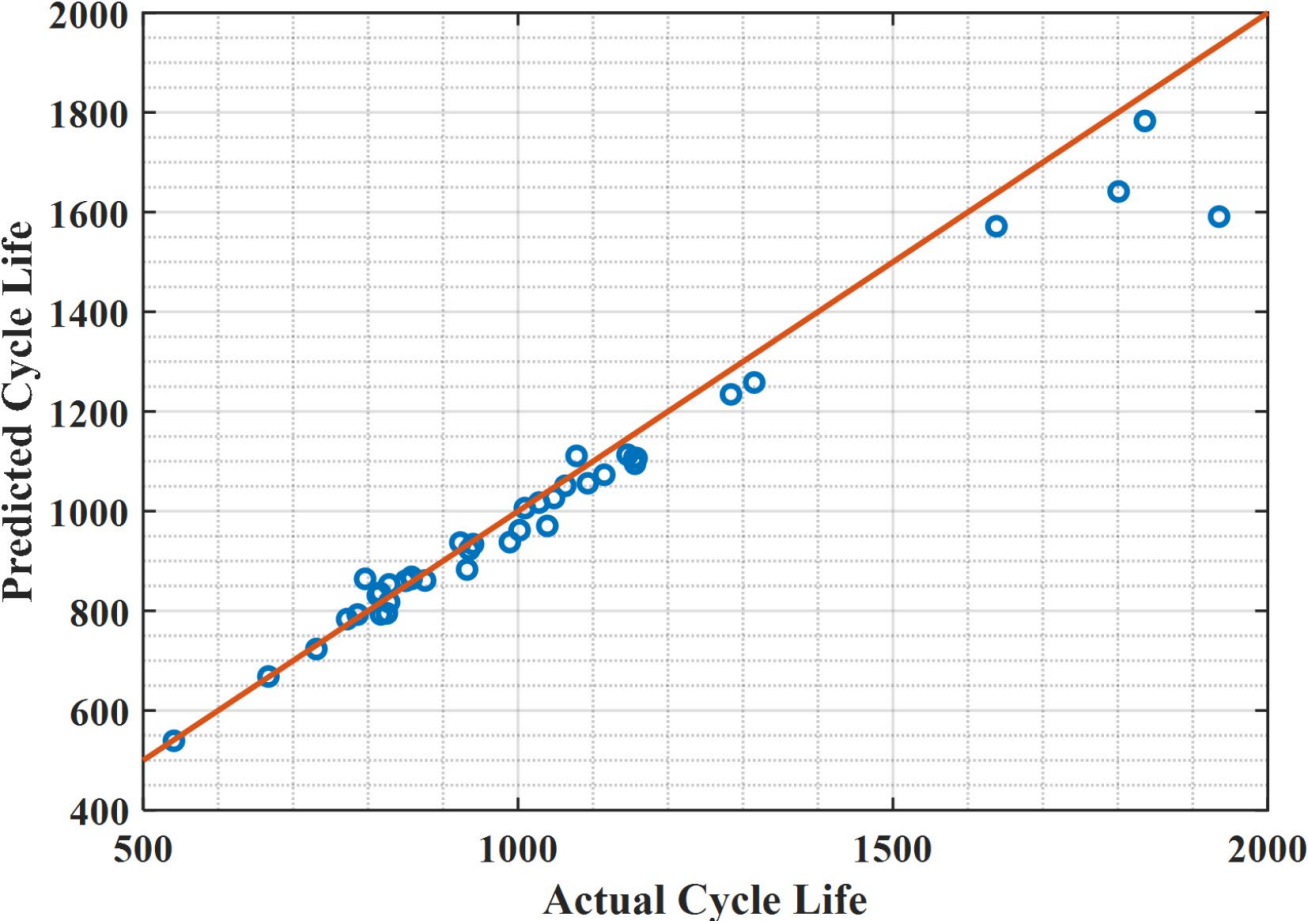
Actual cycle life against predicted cycle life for 40 lithium-ion cells using the data driven with the hybrid methodology.

**Fig. 5:**
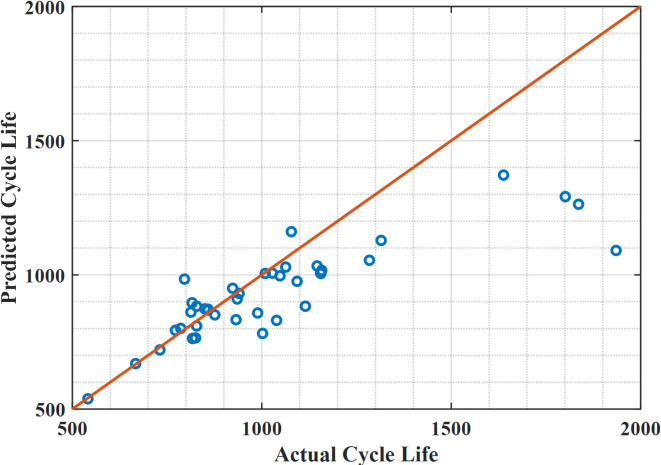
Actual cycle life against predicted cycle life for 40 lithium-ion cells using the linear regression methodology.

**Fig. 6:**
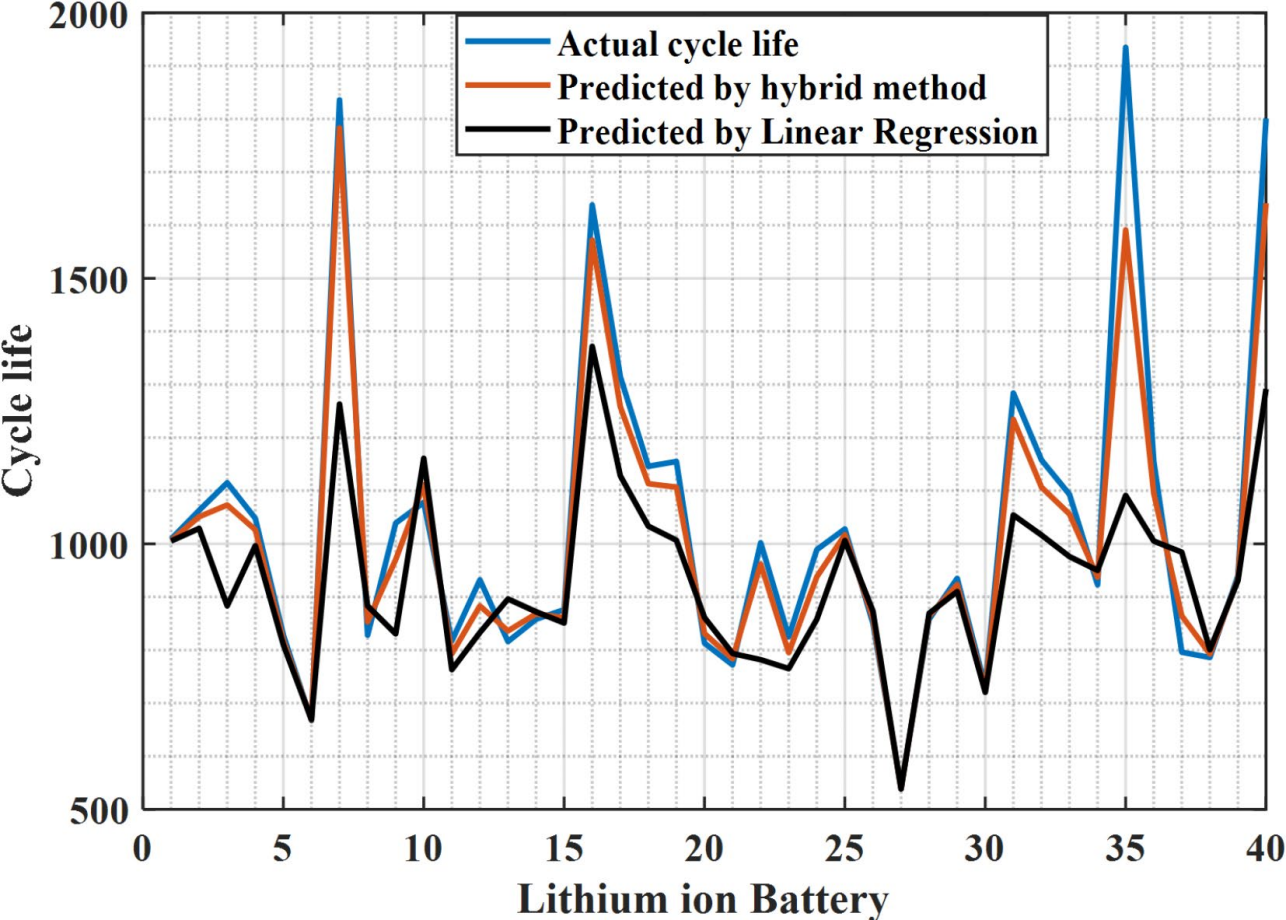
Actual cycle life and predicted cycle life with each individual lithium-ion battery among 40 batteries.

For the 40 lithium-ion batteries used in testing the data, the proposed hybrid extended Kalman filter with Newton Raphson method reduces the difference in error between the predicted data and the actual data of these 40 cells, as shown in Fig. 4, while the linear regression shows a large difference in error as the predicted data is way too far from actual data, which is illustrated in Fig. 5. Figure 6 indicates the actual cycle life and the predicted cycle life using the proposed methodology with each individual lithium-ion battery among 40 batteries. It can be realized that the predicted cycle life using the proposed hybrid extended Kalman filter with Newton Raphson method is very close to the actual cycle life for all 40 batteries. In this regard, the predicted cycle life using the proposed methodology is better than that obtained using the linear regression method for all 40 lithium-ion batteries.

## Conclusion

This paper has introduced a hybrid extended Kalman filter with Newton Raphson method to efficiently predict the lithium-ion battery lifetime. To advance the LIB technology more quickly, its lifetime should be predicted accurately. The precise prediction of LIB lifetime can help in producing new batteries, better use and operation of batteries. It is worthy for noting here that the LIB is a heavy nonlinear system suffering from battery fading, degradation, uncertainty and variability of operating conditions. Therefore, this article has presented a hybrid extended Kalman filter with Newton Raphson method for lifetime prediction of lithium-ion batteries. The data analyses are based on commercial lithium iron phosphate/graphite cells cycled at fast charge. The cycle life expectancy is in the range of 150 to 2,300 cycles. The discharge voltage characteristics are used to present capacity degradation. The effectiveness of the proposed method is verified by making a fair comparison with the linear regression-based machine-learning method. In the testing of 100 lifecycles, the average test error and RMSE record 3.26% and 10.93 compared with the linear regression that achieves 9.1% and 211, respectively. With the combination of extended Kalman filter and Newton Raphson method, the lifetime prediction of LIBs can be further enhanced. The proposed model will be used in the near future in estimating or predicting other quantities in several nonlinear system with uncertainties such as renewable energy, power systems, microgrids and nanogrids, electric vehicles and transportation sectors.

## Data Availability

The datasets used in this study are available at https://data.matr.io/1.
